# Annular Rupture Due to Calcified Nodule Located in Left Ventricular Outflow Tract (LVOT) During Transcatheter Aortic Valve Replacement (TAVR) Managed by Protamine Sulfate

**DOI:** 10.7759/cureus.33417

**Published:** 2023-01-05

**Authors:** Sukhjinder Chauhan, Mina Bhatnagar, Talha N Jilani, Jeffrey Levisman

**Affiliations:** 1 Internal Medicine, Mountainview Hospital, Las Vegas, USA; 2 Internal Medicine, Trinitas Regional Medical Center, Elizabeth, USA; 3 Neurology, Hackensack Meridian Health JFK Medical Center Neuroscience Institute, Edison, USA; 4 Cardiology, Mountainview Hospital, Las Vegas, USA

**Keywords:** tavi, lvot, left ventricular outflow obstruction (lvot), aortic rupture, aortic stenosis (as), annular rupture, self-expanding, balloon-expandable, transcatheter aortic valve repair

## Abstract

Annular rupture is a rare yet fatal complication of transcatheter aortic valve replacement (TAVR). The likelihood of annular rupture is increased by the presence of extensive subannular calcification, excessive balloon dilatation for valve expansion or aggressive valve oversizing to prevent paravalvular leakage during TAVR. Although extensive annular or aortic root calcification increases the likelihood of annular rupture, rupture due to the presence of a calcified nodule in the left ventricular outflow tract (LVOT) is not commonly reported.

We present the case of an 84-year-old man who developed an annular rupture during TAVR, likely due to the presence of a calcified nodule located in LVOT, which was noted on a pre-procedural computed tomography (CT) scan. The rupture was identified early and was successfully reversed with the administration of protamine sulfate during the procedure.

## Introduction

Aortic stenosis is a common valvular heart disease with an estimated prevalence of 3% in adults over the age of 75 years [1]. Transcatheter aortic valve replacement (TAVR) has emerged as a new mainstay for treating this condition. The data collected by the Society of Thoracic Surgeons/American College of Cardiology/Transcatheter Valve Therapy registry (STS/ACC/TVT) showed that in 2019, the number of TAVR procedures (72,991 procedures) exceeded surgical aortic valve replacement (SAVR) procedures (57,626 procedures) in the United States [2]. The increasing use of TAVR is accompanied by a decline in the rate of vascular complications associated with this procedure. Comparing the temporal trends between 2012 and 2013 versus 2014 showed a decrease in vascular complication rates from 5.6% to 4.2% [3]. However, major complications related to this procedure, such as annular rupture, aortic rupture, aortic dissection, and left ventricle perforations, continue to be associated with significant morbidity and mortality. Aortic annular rupture is an uncommon yet serious complication of TAVR and is estimated to occur in almost 0.4-2.3% of cases [4].

In this case report, we present a patient who developed an annular rupture during TAVR and was managed by administering protamine sulfate alone, which subsequently helped seal the rupture during the procedure.

## Case presentation

An 84-year-old man with a past medical history of hypertension and paroxysmal atrial fibrillation on warfarin presented to the hospital with the complaint of “slowing down for the past one to two years.” The patient reported progressive dyspnea on exertion associated with bilateral lower extremity edema. He stated that his dyspnea on exertion has been gradually worsening for the past few months; however, he denied any past episodes of chest pain or syncope. His vital signs were within normal limits. On lung auscultation, mild bibasilar crackles and decreased breath sounds at lung bases were noted. Carotid pulses were delayed bilaterally and a harsh, late-peaking, crescendo-decrescendo murmur was heard at the base of the heart and in the right second intercostal space. Lower extremity 2+ pitting edema was present bilaterally. 

**Figure 1:**
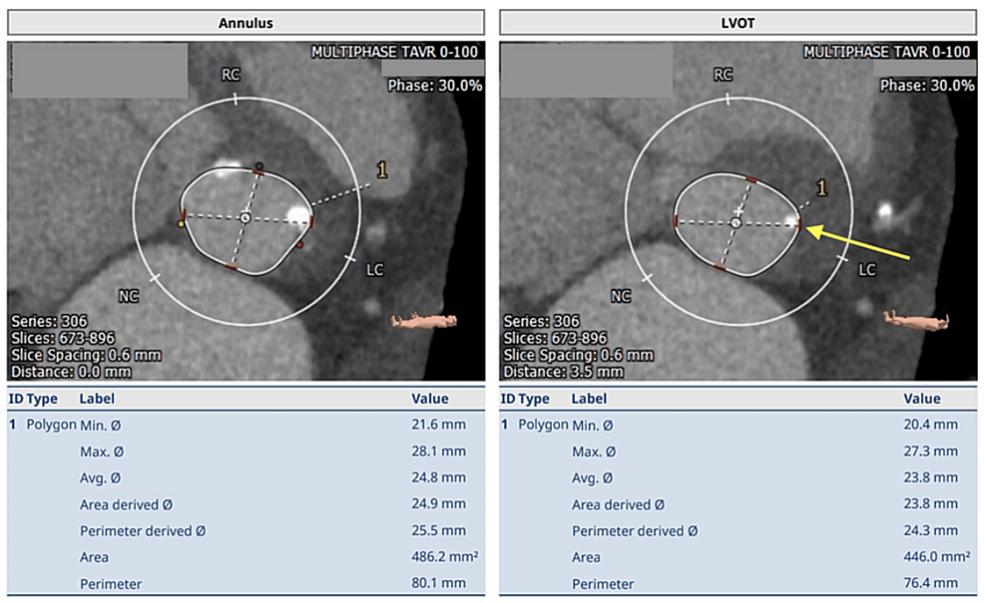
TAVR protocol computed tomography (CT) scans demonstrated no significant aortic annular calcification; however, the presence of a calcified nodule can be seen in the annulus shown in the left panel, which is extending into the LVOT shown in long axis view in the right panel indicated by the yellow arrow. LVOT: left ventricular outflow tract; TAVR: transcatheter aortic valve replacement

An echocardiogram was ordered, which demonstrated an aortic valve area of 0.4 cm^2^, peak aortic jet velocity of 5.56 m/s, transvalvular mean gradient of 77 mm Hg across the aortic valve, left ventricular ejection fraction (LVEF) of 55%, right ventricular systolic pressure (RSVP) of 79 mm Hg, and a mean pulmonary artery pressure of 46 mm Hg. Based on these findings, a diagnosis of symptomatic severe aortic stenosis was established, and aortic valve replacement was recommended. Pre-procedural cardiac catheterization demonstrated right dominant coronary circulation without significant coronary artery disease. TAVR protocol computed tomography (CT) scans demonstrated no significant aortic annular calcification; however, the presence of a calcified nodule could be seen in the annulus shown in Figure 1 (left panel), which extended into the LVOT shown in the long axis view in Figure 1 (right panel), indicated by the yellow arrow.

**Figure 2:**
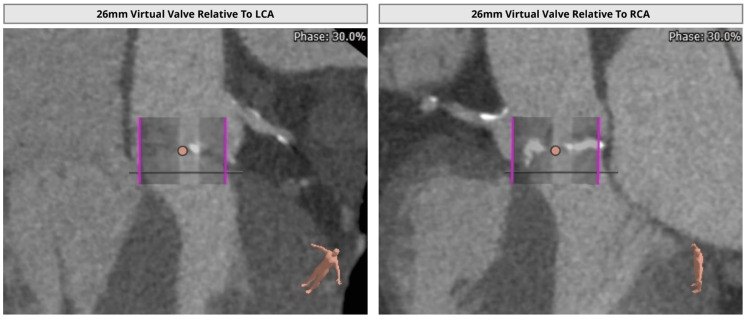
The size of the virtual valve on pre-procedural imaging was measured to be 26 mm. LCA: left coronary artery; RCA: right coronary artery

The size of the virtual bioprosthetic aortic valve on pre-procedural imaging was measured to be 26 mm (Figure 2) indicated by purple lines in two different views.

**Figure 3:**
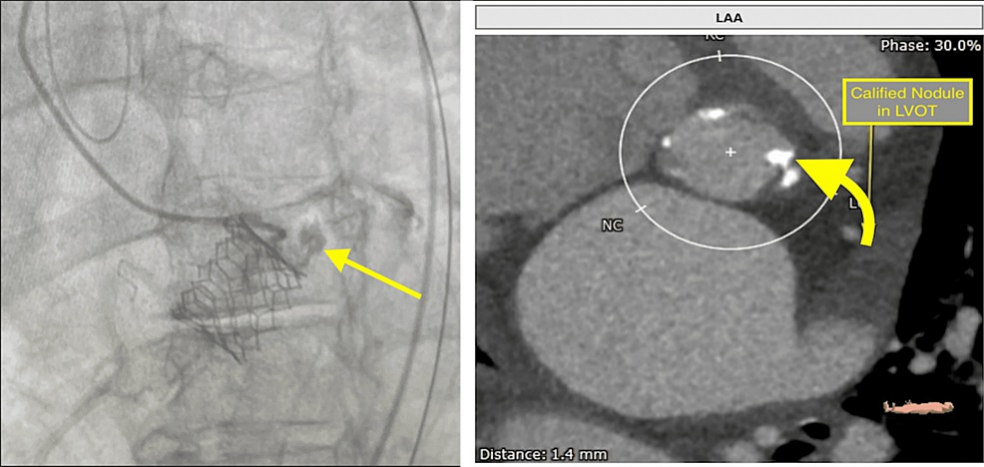
Aortography demonstrates active annular rupture (yellow arrow) in the panel on the left. The calcified nodule in the LVOT (curved yellow arrow) shown in the right panel, is the cause of annular rupture after the deployment of the 26 mm aortic valve. LVOT: left ventricular outflow tract; LAA: left atrial appendage

Following a comprehensive discussion with the heart team (Cardiology, Interventional Cardiology, and Cardiovascular Surgery), balloon-expandable transcatheter aortic valve replacement was planned for the treatment of severe aortic stenosis. During the procedure, the patient was under conscious sedation. A 25 mm balloon was used for pre-dilatation and a 26 mm bioprosthetic valve was successfully deployed. Following the valve deployment, a repeat aortography demonstrated normal left coronary artery (LCA) and right coronary artery (RCA) perfusions; however, the annular rupture was also noted (Figure 3, left panel), originating from LVOT where the calcified nodule was located Figure 3, right panel).

The patient remained hemodynamically stable; however, he reported a new onset of substernal chest pain. Upon quick identification of the rupture, it was decided to administer intravenous protamine sulfate to reverse anticoagulation and to help seal the rupture. Repeat aortography was performed approximately after two minutes, which showed a resolving leak, and again after 10 minutes, which showed no evidence of extravasation. At this time, the patient, under conscious sedation, reported improvement in chest pain. 

The patient was admitted to the intensive care unit (ICU) for close monitoring. On postoperative day 0 in the ICU, he was hemodynamically and clinically stable and did not have any recurrent chest pain. Serial transthoracic echocardiogram (TTE) over the next 24 hours demonstrated no pericardial effusion. On postoperative day 1, the patient remained asymptomatic and was discharged without any further complications. 

## Discussion

There are several predictors for the development of annular rupture during TAVR, including the location and extent of aortic valve calcification, valve oversizing, and the choice of prosthesis. The more extensive the calcification, the higher the risk of rupture. Moreover, calcification close to the muscular LVOT or calcification that has extended into the LVOT in the non-coronary cusp also increases the likelihood of rupture [4]. In a retrospective analysis involving 1, 635 patients undergoing TAVR between 2007 and 2018, annular rupture was found to occur in 10 patients [5]. Eight of these 10 patients had moderate or severe LVOT calcification, while two out of the 10 patients had none or mild calcification. These results showed that annular rupture is more commonly associated with moderate or severe LVOT calcification versus none or mild calcification (2.3% vs 0.2%; p <0.001). In the patients who developed annular rupture, LVOT calcification was found to be present in the free myocardial wall of the left ventricle. Six patients had LVOT calcification in the region below the non-coronary cusp, while three patients had calcification in the interventricular septum [5]. Our patient was also noted to have a calcified nodule in the LVOT on pre-TAVR CT scans. This is an uncommon finding and can easily be overlooked, resulting in the development of annular rupture.

Excessive or aggressive balloon post-dilatation and prosthesis oversizing by over 20% may also contribute to the development of annular rupture [4,6]. The choice of the transcatheter heart valve (THV), i.e., balloon-expandable versus self-expandable, is another significant predictor of this complication during TAVR [4]. BE-THVs have been found to present a higher risk of annular rupture than self-expandable-THVs [7]. However, balloon-expandable-THVs appear to have a competitive edge over SE-THVs when it comes to residual paravalvular regurgitation (PVR) and the ability for easy coronary re-access [8]. Hence, in our patient, the balloon-expandable valve was chosen for TAVR. In addition, we pre-calculated the area of valve expansion and found it to be about 6.4%, which was significantly less than the 20% oversizing that has been reported to be associated with an increased risk of rupture [4,6]. However, despite our careful pre-procedural planning, our patient did develop an annular rupture.

The management of annular rupture during TAVR depends on the size, location of the tear, and the patient's hemodynamic status. Hemodynamics are affected when the rupture extends into the pericardial space leading to cardiac tamponade. Therefore, close observation and a high clinical suspicion for annular rupture should be maintained, especially in patients who experience pericardial irritation or if there is any diagnostic evidence of bleeding into the pericardium post-procedurally. Emergency surgical intervention is required in patients with significant, uncontained rupture resulting in hemodynamic instability. However, in hemodynamically stable patients, a conservative management strategy, including reversal of anticoagulation and close surveillance, may be adequate [4, 9]. Other treatment options include coil embolization, polymer injection, and vascular plugs [10,11].

In our patient, annulus rupture was noted to be originating from LVOT where the calcified nodule was located on the pre-TAVR CT scan (Figure 3, right panel). Our patient remained hemodynamically stable, and we decided to reverse anticoagulation with protamine sulfate to seal the leak prior to attempting coiling. After using protamine sulfate, aortography was performed twice and did not reveal any leakage. It is important to note that protamine sulfate is often administered after TAVR to reduce the risk of bleeding complications, although the impact of protamine sulfate on major bleeding complications has not been found to be significant [12]. However, the administration of protamine sulfate during TAVR led to the successful management of annular rupture in our patient. Therefore, we propose that in addition to coiling and polymer injection, the administration of protamine sulfate may be considered for the management of hemodynamically stable patients with annular rupture during the TAVR procedure. 

## Conclusions

TAVR is being increasingly used for the treatment of symptomatic severe aortic stenosis. However, major complications of this procedure, such as annular rupture, continue to be associated with an increased risk of mortality. The likelihood of annular rupture during TAVR increases with the presence of extensive subannular calcification; however, rarely, this complication may develop secondary to calcified nodules in the LVOT. In patients with annular rupture who remain hemodynamically stable, reversal of anticoagulation by administering protamine sulfate during the procedure can be attempted. Early identification and management of annular rupture during TAVR may play a critical role in lowering the mortality associated with this complication. 
